# Urinary Phosphorus Excretion and Cardiovascular Outcomes in Patients with Pre-Dialysis Chronic Kidney Disease: The KNOW-CKD Study

**DOI:** 10.3390/nu15102267

**Published:** 2023-05-10

**Authors:** Sang Heon Suh, Tae Ryom Oh, Hong Sang Choi, Chang Seong Kim, Eun Hui Bae, Seong Kwon Ma, Kook-Hwan Oh, Young Youl Hyun, Suah Sung, Soo Wan Kim

**Affiliations:** 1 Department of Internal Medicine, Chonnam National University Medical School, Gwangju 61469, Republic of Korea; 2 Department of Internal Medicine, Chonnam National University Hospital, Gwangju 61469, Republic of Korea; 3 Department of Internal Medicine, Seoul National University Hospital, Seoul 03080, Republic of Korea; 4 Department of Internal Medicine, Kangbuk Samsung Hospital, Sungkyunkwan University School of Medicine, Seoul 03181, Republic of Korea; 5 Department of Internal Medicine, Eulji Medical Center, Eulji University, Seoul 01830, Republic of Korea

**Keywords:** 24 h urine, cardiovascular outcome, chronic kidney disease, major adverse cardiac event, phosphorus

## Abstract

The relationship between 24-h urinary phosphorus excretion (24 h UPE) and cardiovascular disease in patients with pre-dialysis chronic kidney disease (CKD) has rarely been studied, despite the fact that the relationship between serum phosphorus level and the risk of a cardiovascular event is well established. A total of 1701 patients with pre-dialysis CKD were finally included for the analyses and were divided into tertiles by 24 h UPE (first tertile (T1, 349.557 (mean) ± 88.413 (standard deviation)), second tertile (T2, 557.530 ± 50.738), and third tertile (T3, 851.695 ± 171.593). The study outcome was a six-point major adverse cardiac event (MACE). The median follow-up duration was 7.992 years. Kaplan–Meier curve analysis visualized that the cumulative incidences of a six-point MACE (*p* = 0.029) significantly differed from 24 h UPE levels, as the incidence rate of the study outcomes was highest in T1 and lowest in T3. Cox proportional hazard models unveiled that, compared to T1, the risk of a six-point MACE was significantly decreased in T3 (adjusted hazard ratio (HR) 0.376, 95% confidence interval (CI) 0.207 to 0.683). The restricted cubic spline curve analysis visualized an inverted S-shaped association between 24 h UPE level and the risk of a six-point MACE, indicating a significantly increased risk of a six-point MACE in patients with a low 24 h UPE level. In conclusion, low 24 h UPE is associated with adverse cardiovascular outcomes in patients with CKD. Our finding emphasizes that low 24 h UPE should not be a reliable marker for dietary restriction of phosphorus that essentially leads to better outcomes in patients with CKD.

## 1. Introduction

The kidney is a major organ for the regulation of phosphorus homeostasis [1,2,3]. The excretory capacity of the kidney for phosphorus is limited at relatively early stages of CKD despite the hormonal compensation of parathyroid hormone (PTH) and fibroblast growth factor 23 (FGF23) [3,4,5], contributing to the elevation of serum phosphorus level (i.e., hyperphosphatemia). Regarding clinical outcomes, hyperphosphatemia in individuals with CKD is especially concerning. The relationship between serum phosphorus level and the chance of cardiovascular event (CVE) or cardiovascular mortality among patients with end-stage renal disease (ESRD) has long been well established [6]. Even in patients with CKD, elevated serum phosphorus levels have been linked to vascular calcification [7,8], arterial stiffness [9,10,11], and incident CVE [12,13,14].

As dietary phosphorus restriction is a critical strategy to avoid hyperphosphatemia in patients with CKD [15,16,17], 24-h urinary phosphorus excretion (24 h UPE) has been implemented in previous studies to estimate the intestinal absorption of dietary phosphorus [18,19], In this regard, it is surprising that the relationship between 24 h UPE and the risk of CVE in patients with CKD has received so little attention [20] because, if 24 h UPE is a reliable indicator of intestinal absorption of dietary phosphorus, a high 24 h UPE as a result of a high dietary phosphorus load could be assumed to ultimately increase the risk of CVE.

A recent study in patients with CKD, however, revealed that 24 h UPE is highly variable despite a tightly controlled dietary loading of phosphorus, and that 24 h UPE is not well correlated with dietary loading or intestinal absorption, but is instead inversely associated with net phosphorus retention [21]. Based on the inverse correlation between 24 h UPE and net phosphorus retention, the clinical context of 24 h UPE could completely differ from the aforementioned postulation. This is because low 24 h UPE may be linked to adverse CV outcomes in patients with CKD by potentially contributing to hyperphosphatemia by phosphorus retention [20,21].

Therefore, taking advantage of a large-sized (*n* = 1701), long-term cohort (median duration of follow-up 7.992 years), the purpose of the current study was to examine the association between 24 h UPE and the risk of CVE in patients with CKD. We here hypothesized that low 24 h UPE may be associated with adverse CV outcomes, a six-point major adverse cardiac event (MACE), in patients with CKD.

## 2. Materials and Methods

### 2.1. Study Design

**Figure 1:**
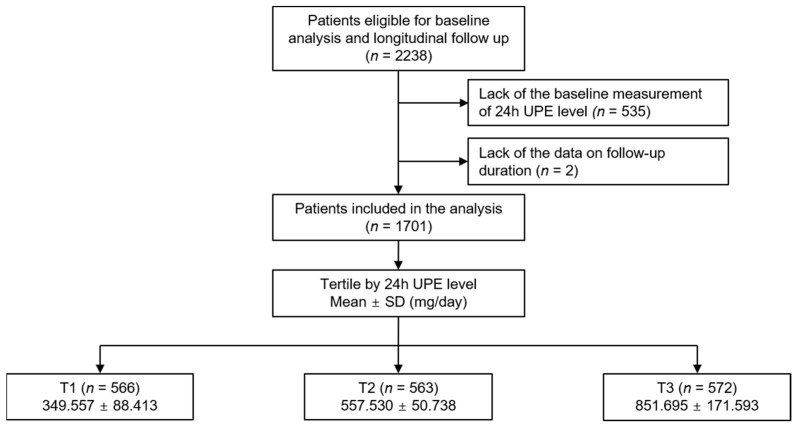
Flow diagram of the study participants. Abbreviations: 24 h UPE, 24 h urinary phosphorus excretion; T1, 1st tertile; T2, 2nd tertile; T3, 3rd tertile.

The Korean Cohort Study for Outcomes in Patients With Chronic Kidney Disease (KNOW-CKD) (NCT01630486 at http://www.clinicaltrials.gov; accessed on 5 June 2019) [22] involves nine tertiary-care general hospitals across the country. The Declaration of Helsinki’s guiding principles were followed in conducting the study. Each participating center’s Institutional Review Board gave its approval to the study protocol. Patients between the ages of 20 and 75 with CKD stages 1 to pre-dialysis stages 5 were enrolled from 2011 to 2016. Throughout the follow-up period, each subject willingly gave their informed consent and was under close observation. Each participating center kept track of the study outcomes. The correctness of the study outcomes was double-checked by the cooperating investigators for each incident. A total of 1701 patients were eventually included and analyzed after excluding those without initial measurements of 24 h UPE (*n* = 535) and those without data on follow-up duration (*n* = 2) (Figure 1). The study observation period ended on 31 March 2022. The median follow-up duration was 7.992 years.

### 2.2. Data Collection from Participants

Demographic data includes information on age, sex, comorbidity indexed by Charlson comorbidity index score, the primary cause of CKD, smoking status, and medication history (including diuretics, anti-HTN drugs, statins, and ACEi/ARBs). The participants’ height, weight, and waist-to-hip ratio (WHR) were all measured by skilled staff members. The body mass index (BMI) was calculated as the weight divided by the square of the height. Systolic and diastolic blood pressure (SBP and DBP) were measured with an electronic sphygmomanometer following 5 min of seated relaxation. Venous samples were collected for laboratory tests, such as creatinine (Cr), after an overnight fast. Estimated glomerular filtration rate (eGFR) was computed using the serum Cr level and the Chronic Kidney Disease Epidemiology Collaboration equation [23]. Urine spot samples taken at random, preferably after the initial void, were tested for the albumin-to-Cr ratio (ACR). In order to determine 24 h urine protein excretion (UPE) as well as 24 h urinary creatinine excretion, 24 h urinary samples were also taken at the baseline. Cardiologists at the participating hospitals performed complete two-dimensional M-mode and Doppler examinations using conventional techniques while being blinded to the clinical information. An M-mode evaluation was finished per the advice of the American Society of Echocardiography [24]. Devereux formula was used to determine left ventricular (LV) mass. Normalizing LV mass to height^2^ (g/m^2^) led to the calculation of the LV mass index (LVMI) [24].

### 2.3. Exposure and Study Outcome

The study participants were divided into tertiles by 24 h UPE (1st tertile (T1), 2nd tertile (T2), and 3rd tertile (T3)) (Figure 1). The primary outcome of interest was a 6-point MACE, which was defined as death from CV causes and any CV events including non-fatal acute myocardial infarction and unstable angina requiring percutaneous coronary artery intervention or coronary bypass surgery, ischemic or hemorrhagic stroke, congestive heart failure, and symptomatic arrhythmia requiring hospitalization [25].

### 2.4. Statistical Analysis

One-way analysis of variance and the χ^2^ test were used for continuous and categorical variables, respectively, to assess the baseline characteristics by 24 h UPE level. The cumulative incidence of the outcome events was represented using Kaplan–Meier curves, and the data was assessed using the log-rank test. Individuals with any missing data were eliminated from further investigation. In order to evaluate the relationship between the 24 h UPE level and the likelihood of CVE, Cox proportional hazard regression models were employed. Patients who were lost to follow-up were censored based on their last scheduled appointment. The following variables were adjusted using models. Crude hazard ratios (HRs) are represented by Model 1. Age, gender, Charlson comorbidity index, primary CKD cause, smoking status, medication (ACEi/ARBs, diuretics, number of anti-HTN medications, statins), WHR, and SBP were all adjusted in Model 2. Hemoglobin, albumin, total calcium, inorganic phosphorus, total cholesterol, low-density lipoprotein cholesterol (LDL-C), high-density lipoprotein cholesterol (HDL-C), triglycerides (TG), fasting glucose, 25(OH)D, high-sensitivity C-reactive protein (hs-CRP), eGFR, and spot urine ACR were also adjusted in Model 3. Additional LVMI and LVEF adjustments were made to Model 4. Hazard ratios (HRs) and 95% confidence intervals (CIs) were used to present the findings of Cox proportional hazard models. Using restricted cubic spline curves, the link between the risk of CVE and the 24 h UPE level (a continuous variable) was assessed. We performed numerous sensitivity analyses in order to validate our conclusions. First, we looked at whether there was a relationship between the 24 h UPE level and the risk of a 3-point MACE, which was defined as a CV death, a non-fatal myocardial infarction, or a non-fatal stroke [26], or a 4-point MACE, which was defined as a death from a CV cause and any CV events, including non-fatal acute myocardial infarction, unstable angina receiving percutaneous coronary artery intervention. Second, we excluded participants with CKD stage 1 because they are believed to have kidney function that is close to normal and may not truly represent the CKD population. Third, we omitted participants with CKD stage 5 since they are relatively small in number and may overestimate the association between the 24 h UPE level and the probability of CVE due to far advanced CKD. Fourth, we performed another round of Cox regression analysis with multiple imputation. Age, gender, BMI, eGFR, and spot urine ACR were used as the defining characteristics for the pre-specified subgroups in the analyses we carried out. These investigations sought to ascertain whether the clinical context has an impact on the relationship between 24 h UPE level and the risk of CVE. Statistics were regarded as significant when two-sided *p* values were less than 0.05. R (version 4.1.1; R Project for Statistical Computing, Vienna, Austria) and SPSS for Windows version 22.0 (IBM Corp., Armonk, NY, USA) were used to conduct the statistical analysis.

## 3. Results

### 3.1. Baseline Characteristics

The baseline characteristics of the study participants were described according to 24 h UPE levels (Table 1). The mean duration of follow-up was longest in T3 and shortest in T1. The mean age was highest in T1 and lowest in T3. The proportion of male participants was highest in T3 and lowest in T1. The proportion of the subject with Charlson comorbidity index ≥ 3 and diabetes mellitus was significantly higher in T1. The proportion of smokers was significantly higher in T3. Although the frequency of ACEi/ARB medication was marginally higher in T3 (*p* = 0.044), that of diuretic use or medication for no less than three anti-hypertensive drugs was significantly higher in T1. BMI, WHR, and DBP were significantly higher in T3. Hemoglobin, albumin, total calcium, total cholesterol, LDL-C, and 25(OH)D levels were also significantly higher in T3, whereas serum phosphorus level was highest in T1. Spot urine ACR and serum creatinine level were significantly higher in T1. Accordingly, mean eGFR was significantly lower in T1. The incidence of a six-point MACE also significantly differed from the baseline CKD stages (Table S1). The echocardiographic findings of study participants were also summarized by 24 h UPE level (Table S2). LVMI was marginally higher in T3 (*p* = 0.048), while E/e’ was highest in T1. Left atrial diameter and the frequency of valve calcification were significantly higher in T1, whereas posterior wall thickness, interventricular wall thickness, LV end-diastolic diameter, and LV end-systolic diameter were significantly higher in T3.

### 3.2. Association between 24 h UPE Level and the Risk of CVE in Patients with CKD

**Figure 2:**
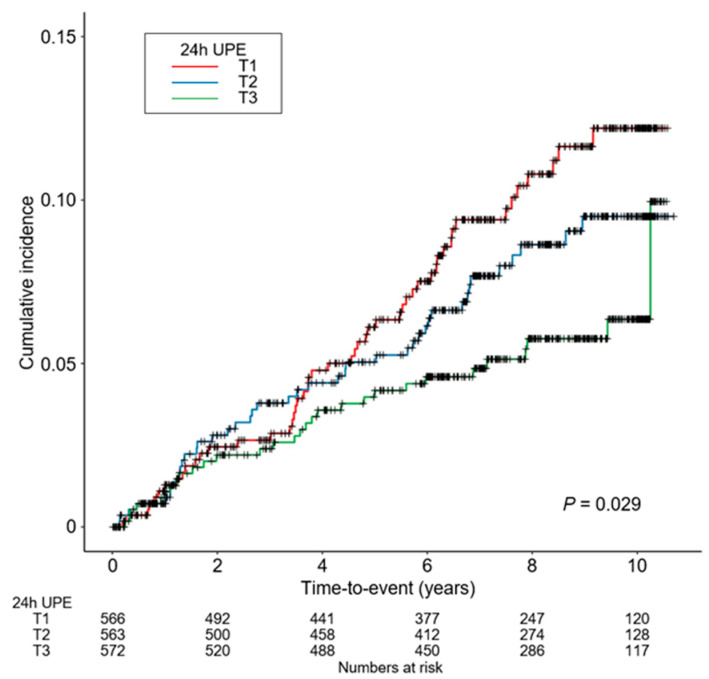
Kaplan–Meier survival curve for cumulative incidence of 6-point MACE by 24 h UPE. *p* value by Log-rank test. Abbreviations: 24 h UPE, 24 h urinary phosphorus excretion; T1, 1st tertile; T2, 2nd tertile, T3, 3rd tertile.

**Figure 3:**
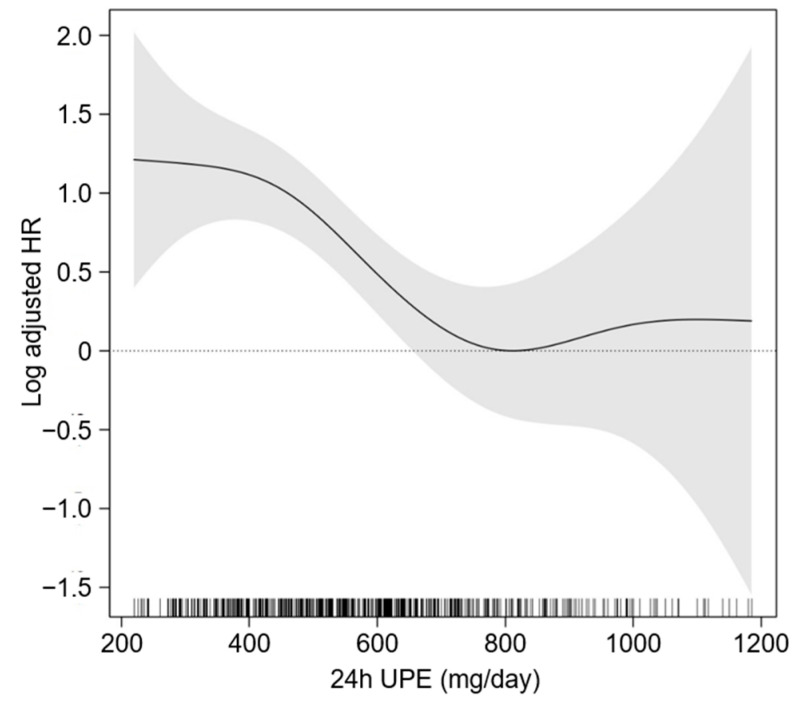
Restricted cubic spline of 24 h UPE on 6-point MACE. Adjusted HR of 24 h UPE as a continuous variable for 6-point MACE is depicted. The model was adjusted for age and sex, age-adjusted Charlson comorbidity index, the main cause of CKD, smoking status, medication (ACEIs/ARBs, diuretics, number of anti-HTN drugs, statins), WHR, SBP, hemoglobin, albumin, total calcium, phosphorus, fasting glucose, hs-CRP, eGFR, spot urine ACR, LVMI, and LVEF. Abbreviations: 24 h UPE, 24 h urinary phosphorus excretion; HR, hazard ratio.

To compare the cumulative incidence of study outcomes by 24 h UPE levels, Kaplan–Meier curves were analyzed (Figure 2). The cumulative incidences of both six-point MACEs (*p* = 0.029) significantly differed from 24 h UPE levels, as the incidence rate of the study outcomes was highest in T1 and lowest in T3. To assess the independent association between 24 h UPE level and the risk of CVE, Cox proportional hazard models were analyzed (Table 2). Compared to T1, the risk of a six-point MACE was significantly decreased in T3 (adjusted HR 0.376, 95% CI 0.207 to 0.683). In the restricted cubic spline curve analysis, an inverted S-shaped association between 24 h UPE level and the risk of -point MACE was observed, indicating the significantly increased risk of a six-point MACE in patients with low 24 h UPE levels (Figure 3).

### 3.3. Sensitivity Analysis

The analyses of Cox proportional hazard models demonstrated that high 24 h UPE (i.e., T3) is also associated with a lower risk of three-point (adjusted HR 0.436, 95% CI 0.213 to 0.893) or four-point (adjusted HR 0.492, 95% CI 0.257 to 0.942) MACE (Table S3). After excluding the patients at CKD stage 1, high 24 h UPE was still associated with a lower risk of a six-point MACE (adjusted HR 0.415, 95% CI 0.227 to 0.706) (Table S4). The association between high 24 h UPE and a six-point MACE (adjusted HR 0.436, 95% CI 0.213 to 0.893) remained robust even after excluding the patients at CKD stage 5 (Table S5). Even with the changes In the covariables in the model, the association between 24 h UPE levels and the risk of CVE remained robust (Table S6). Finally, after replacing the missing values by multiple imputation, high 24 h UPE remained significantly associated with a lower risk of a six-point MACE (adjusted HR 0.534, 95% CI 0.323 to 0.882) (Table 3).

### 3.4. Prespecified Subgroup Analysis

We conducted a series of prespecified subgroup analyses to examine whether the relation between 24 h UPE levels and the risk of CVE is modified by clinical factors. The association between 24 h UPE level and the risk of CVE was more prominently observed in the patients with age < 60 years (*p* for interaction = 0.025), although gender, obesity (BMI < 23 kg/m^2^ vs. BMI ≥ 23 kg/m^2^), kidney function (eGFR ≥ 45 mL/min./1.73 m^2^ vs. eGFR < 45 mL/min./1.73 m^2^), or albuminuria (spot urine ACR < 300 mg/g vs. spot urine ACR ≥ 300 mg/g) did not significantly modify the association (Table 4).

## 4. Discussion

In the current study, we demonstrated that low 24 h UPE is associated with adverse CV outcomes in patients with CKD. We prove the robustness of our findings via a series of sensitivity analyses, including multiple imputation. We also found that the association between 24 h UPE level and the risk of CVE is more prominently observed in patients with age < 60 years.

Our finding is clinically critical because 24 h UPE has been previously assumed as an index of intestinal absorption of dietary phosphorus [18,19]. Based on the assumption, it seems reasonable that high 24 h UPE as a reflection of high dietary loading of phosphorus should ultimately increase the risk of CVE, which is contradictory to the major finding of the current study. Thus, we believe that the current study presents a proof-of-concept finding to prevent the misinterpretation of 24 h UPE in the context of the risk of CVE in patients with CKD and emphasize that low 24 h UPE should not be a reliable marker for dietary restriction of phosphorus that essentially leads to better outcomes in patients with CKD.

The association between low 24 h UPE and the risk of CVE has been once previously reported [20], where high 24 h UPE decreased the risk of incident CVE. The study included a total of 880 patients with stable CV disease with normal kidney function to ‘moderate’ CKD; hence, the mean eGFR of the participants was relatively high (71 mL/min/1.73 m^2^), and serum phosphorus level was not different from 24 h UPE level [20]. In contrast, the current study included almost twice as many patients as the previous study, with CKD stage 1 to pre-dialysis stage 5. Accordingly, the mean eGFR of the participants in the present study (50 mL/min/1.73 m^2^) was substantially lower than that of the previous study. In concordance with our findings, an impaired phosphaturic response to FGF23 with low fractional excretion of phosphorus to FGF23 ratio was associated with severe abdominal aortic calcification among patients with CKD stages 3 to 4 [27]. Therefore, the current study confirms the association between low 24 h UPE and adverse CV outcomes and expands the finding to patients with more advanced CKD.

This unexpected association could be primarily attributed to the inverse correlation between 24 h UPE and net phosphorus retention in patients with CKD [21]. In a previous study that exposed a total of eight patients with CKD to tightly controlled phosphorus intake, 24 h UPE was not significantly correlated with dietary phosphorus loading or intestinal absorption [21]. Rather, the authors reported that, via quantitative analyses, 24 h UPE negatively correlated with whole-body retention of phosphorus [21]. We also observed an inverse correlation between serum phosphorus level and 24 h UPE in the current study (Table 1), suggesting net phosphorus retention among patients with relatively low 24 h UPE levels.

Yet, we still cannot completely exclude the possibility that low 24 h UPE may indicate protein malnutrition that is closely related to adverse CV outcomes in patients with CKD [28,29]. Although dietary restriction of phosphorus is commonly associated with improved outcomes in patients with CKD [30,31], dietary phosphorus intake is usually closely linked to protein intake, suggesting low 24 h UPE might be a result of inadequately low amount of protein calorie [17]. Indeed, nutritional indices, such as serum albumin and total cholesterol levels, were significantly lower in the subjects with low 24 h UPE levels (Table 1). Therefore, the association of 24 h UPE with CV outcomes in the context of nutritional status should be further elucidated among patients with CKD.

We acknowledge some limitations of the present study. First, we cannot present confirmatory data on the causal relation between low 24 h UPE and the risk of CVE due to the observational nature of the study. However, a similar result from the patients with moderate CKD has been previously reported [20], supporting the association between low 24 h UPE and the risk of CVE in patients with CKD. Second, we are not able to prove a precise mechanism that links low 24 h UPE and higher risk of CVE among patients with CKD. However, evidence so far suggests that net phosphorus retention [21] and/or malnutrition–inflammation [17,28] in relation to low 24 h UPE could possibly contribute to the increased risk of CVE among patients with CKD. Third, 24 h UPE levels were determined only once at the baseline, while the urinary excretion of phosphorus should be variable day by day, which could be a reason why we are not able to confirm the association between 24 h UPE and the risk of CVE based on the current study. Lastly, because all of the study participants in the present study are limited to Koreans residing in South Korea, the extrapolation of the result of the study to the other populations requires a precaution. Nevertheless, similar results were previously reported from a study conducted in the United States [20].

In conclusion, we report that low 24 h UPE is associated with adverse CV outcomes in patients with CKD. Our findings emphasize that low 24 h UPE should not be a reliable marker for dietary restriction of phosphorus that essentially leads to better outcomes in patients with CKD.

## Figures and Tables

**Table 1:** Baseline characteristics of study participants by 24 h UPE.

		24 h UPE		*p* Value
	T1	T2	T3	
Follow-up duration (year)	6.908 ± 3.050	7.259 ± 2.814	7.382 ± 2.747	0.019
Age (year)	55.981 ± 12.021	54.128 ± 12.030	52.395 ± 12.159	<0.001
Male	249 (44.0)	350 (62.2)	434 (75.9)	<0.001
Charlson comorbidity index				<0.001
0–3	368 (65.0)	387 (68.7)	449 (78.5)	
4–5	187 (33.0)	165 (29.3)	115 (20.1)	
6–7	11 (1.9)	10 (1.8)	8 (1.4)	
≥8	0 (0.0)	1 (0.2)	0 (0.0)	
Primary cause of CKD				<0.001
DM	172 (30.4)	153 (27.2)	109 (19.1)	
HTN	103 (18.2)	112 (19.9)	129 (22.6)	
GN	149 (26.3)	151 (26.8)	195 (34.2)	
TID	2 (0.4)	3 (0.5)	5 (0.9)	
PKD	95 (16.8)	120 (21.3)	93 (16.3)	
Others	45 (8.0)	24 (4.3)	40 (7.0)	
Smoking status				<0.01
Non-smoker	361 (63.8)	292 (51.9)	234 (41.0)	
Ex-smoker	60 (10.6)	81 (14.4)	115 (20.1)	
Current smoker	145 (25.6)	190 (33.7)	222 (38.9)	
Medication				
ACEi/ARBs	480 (84.8)	479 (85.1)	511 (89.3)	0.044
Diuretics	211 (37.3)	165 (29.3)	161 (28.1)	0.002
Anti-HTN drugs ≥ 3	202 (35.7)	161 (28.6)	170 (29.7)	0.022
Statins	296 (52.3)	289 (51.3)	306 (53.5)	0.765
BMI (kg/m^2^)	23.846 ± 3.487	24.430 ± 3.275	25.743 ± 3.263	<0.001
Waist-to-hip ratio	0.893 ± 0.068	0.900 ± 0.065	0.908 ± 0.057	<0.001
SBP (mmHg)	126.931 ± 17.369	127.652 ± 15.011	128.534 ± 15.096	0.245
DBP (mmHg)	75.342 ± 11.553	77.066 ± 10.517	78.545 ± 10.886	<0.001
Laboratory findings				
Hemoglobin (g/dL)	11.933 ± 1.761	12.840 ± 1.988	13.721 ± 1.840	<0.001
Albumin (g/dL)	4.144 ± 0.427	4.213 ± 0.424	4.274 ± 0.403	<0.001
Total calcium (mg/dL)	9.048 ± 0.612	9.135 ± 0.497	9.174 ± 0.478	0.001
Phosphorus (mg/dL)	3.855 ± 0.732	3.702 ± 0.665	3.540 ± 0.555	<0.001
Total cholesterol (mg/dL)	169.637 ± 38.741	173.141 ± 39.863	176.114 ± 37.416	0.017
HDL-C (mg/dL)	47.919 ± 15.018	49.451 ± 15.015	49.792 ± 15.398	0.088
LDL-C (mg/dL)	92.842 ± 31.764	95.479 ± 31.351	97.806 ± 30.134	0.027
TG (mg/dL)	151.839 ± 94.815	156.248 ± 101.444	163.737 ± 100.745	0.124
Fasting glucose (mg/dL)	109.851 ± 38.327	108.729 ± 32.560	111.614 ± 42.695	0.442
25(OH)D	16.947 ± 7.989	17.226 ± 7.236	18.156 ± 7.335	0.019
hs-CRP (mg/dL)	0.600 [0.200, 1.840]	0.600 [0.200, 1.500]	0.765 [0.300, 1.800]	0.492
Spot urine ACR (mg/g)	391.176 [100.859, 1143.000]	329.409 [50.776, 991.994]	298.723 [57.699, 862.463]	0.002
Creatinine (mg/dL)	2.131 ± 1.376	1.751 ± 1.013	1.498 ± 0.762	<0.001
eGFR (mL/min./1.73 m^2^)	41.066 ± 26.853	51.454 ± 30.055	59.563 ± 29.716	<0.001
CKD stages				<0.001
Stage 1	54 (9.5)	94 (16.7)	135 (23.6)	
Stage 2	75 (13.3)	112 (19.9)	129 (22.6)	
Stage 3a	82 (14.5)	86 (15.3)	113 (19.8)	
Stage 3b	123 (21.7)	127 (22.6)	116 (20.3)	
Stage 4	164 (29.0)	117 (20.8)	72 (12.6)	
Stage 5	68 (12.0)	27 (4.8)	7 (1.2)	

Note: Values for categorical variables are given as number (percentage); values for continuous variables, as mean ± standard deviation or median [interquartile range]. Abbreviations: 24 h UPE, 24 h urinary phosphorus excretion; 25(OH)D, 25-hydroxyvitamin D; ACEi, angiotensin converting enzyme inhibitors; ACR, albumin-to-creatinine ratio; ARBs, angiotensin receptor blockers; BMI, body mass index; CKD, chronic kidney disease; DBP, diastolic blood pressure; DM, diabetes mellitus; eGFR, estimated glomerular filtration rate; GN, glomerulonephritis; HDL-C, high-density lipoprotein cholesterol; hs-CRP, high-sensitivity C-reactive protein; HTN, hypertension; LDL-C, low-density lipoprotein cholesterol; PKD, polycystic kidney disease; SBP, systolic blood pressure; T1, 1st tertile; T2, 2nd tertile; T3, 3rd tertile; TG, triglyceride; TID, tubulointerstitial disease.

**Table 2:** HRs for study outcomes by 24 h UPE level.

	24 h UPE Level	Events, *n* (%)	Model 1		Model 2		Model 3		Model 4	
			HR (95% CIs)	*p* Value	HR (95% CIs)	*p* Value	HR (95% CIs)	*p* Value	HR (95% CIs)	*p* Value
6-point MACE	T1	69 (12.2)	Reference		Reference		Reference		Reference	
	T2	59 (10.5)	0.741 (0.470, 1.169)	0.198	0.804 (0.524, 1.233)	0.317	0.843 (0.532, 1.335)	0.467	0.755 (0.464, 1.229)	0.259
	T3	51 (8.9)	0.417 (0.242, 0.716)	0.002	0.559 (0.343, 0.910)	0.019	0.468 (0.271, 0.808)	0.006	0.376 (0.207, 0.683)	0.001

Note: Model 1, unadjusted model. Model 2, Model 1 + adjusted for age, sex, Charlson comorbidity index, the main cause of CKD, smoking history, medication (ACEi/ARBs, diuretics, number of anti-HTN drugs, statins), WHR, and SBP. Model 3, Model 2 + adjusted for hemoglobin, albumin, total calcium, phosphorus, total cholesterol, LDL-C, HDL-C, TG, fasting glucose, 25(OH)D, hs-CRP, eGFR and spot urine ACR. Model 4, Model 3 + LVMI and LVEF at the baseline. Abbreviations: 24 h UPE, 24 h urinary phosphorus excretion; MACE, major adverse cardiac event; T1, 1st tertile; T2, 2nd tertile; T3, 3rd tertile.

**Table 3:** HRs for study outcomes by 24 h UPE level using multiple imputation.

	24 h UPE Level	Model 1		Model 2		Model 3		Model 4	
		HR (95% CIs)	*p* Value	HR (95% CIs)	*p* Value	HR (95% CIs)	*p* Value	HR (95% CIs)	*p* Value
6-point MACE	T1	Reference		Reference		Reference		Reference	
	T2	0.803 (0.533, 1.210)	0.297	0.817 (0.536, 1.247)	0.352	0.823 (0.533, 1.270)	0.380	0.818 (0.529, 1.265)	0.369
	T3	0.545 (0.346, 0.857)	0.010	0.563 (0.346, 0.914)	0.022	0.531 (0.321, 0.878)	0.016	0.534 (0.323, 0.882)	0.016

Note: Model 1, unadjusted model. Model 2, Model 1 + adjusted for age, sex, Charlson comorbidity index, the main cause of CKD, smoking history, medication (ACEi/ARBs, diuretics, number of anti-HTN drugs, statins), WHR, and SBP. Model 3, Model 2 + adjusted for hemoglobin, albumin, total calcium, phosphorus, total cholesterol, LDL-C, HDL-C, TG, fasting glucose, 25(OH)D, hs-CRP, eGFR and spot urine ACR. Model 4, Model 3 + LVMI and LVEF at the baseline. Abbreviations: 24 h UPE, 24 h urinary phosphorus excretion; MACE, major adverse cardiac event; T1, 1st tertile; T2, 2nd tertile; T3, 3rd tertile.

**Table 4:** HRs for the 6-point MACE by 24 h UPE level in various subgroups.

	24 h UPE Level	Events, *n* (%)	Unadjusted HR (95% CIs)	*p* for Interaction	Adjusted HR (95% CIs)	*p* for Interaction
Age < 60 years	T1	23 (7.0)	Reference	0.04	Reference	0.025
	T2	17 (4.9)	0.705 (0.376, 1.319)		0.348 (0.147, 0.822)	
	T3	8 (2.0)	0.279 (0.125, 0.623)		0.141 (0.046, 0.435)	
Age ≥ 60 years	T1	27 (11.3)	Reference		Reference	
	T2	25 (11.5)	0.915 (0.531, 1.578)		1.047 (0.547, 2.005)	
	T3	22 (12.4)	0.956 (0.544, 1.679)		0.620 (0.295, 1.301)	
Male	T1	32 (12.9)	Reference	0.476	Reference	0.930
	T2	30 (8.6)	0.602 (0.366, 0.991)		0.733 (0.404, 1.331)	
	T3	27 (6.2)	0.424 (0.254, 0.708)		0.376 (0.192, 0.738)	
Female	T1	18 (5.7)	Reference		Reference	
	T2	12 (5.6)	0.972 (0.468, 2.019)		0.971 (0.384, 2.457)	
	T3	3 (2.2)	0.348 (0.103, 1.183)		0.216 (0.040, 1.177)	
BMI < 23 kg/m^2^	T1	26 (10.3)	Reference	0.341	Reference	0.856
	T2	14 (7.3)	0.663 (0.346, 1.270)		0.826 (0.302, 2.261)	
	T3	4 (3.7)	0.305 (0.107, 0.875)		0.337 (0.081, 1.396)	
BMI ≥ 23 kg/m^2^	T1	24 (7.7)	Reference		Reference	
	T2	28 (7.6)	0.955 (0.554, 1.647)		0.791 (0.412, 1.519)	
	T3	26 (5.6)	0.696 (0.399, 1.212)		0.406 (0.192, 0.860)	
eGFR ≥ 45 mL/min./1.73 m^2^	T1	18 (9.1)	Reference	0.028	Reference	0.261
	T2	23 (8.3)	0.865 (0.467, 1.604)		0.779 (0.349, 1.738)	
	T3	11 (3.1)	0.320 (0.151, 0.677)		0.229 (0.081, 0.653)	
eGFR < 45 mL/min./1.73 m^2^	T1	32 (8.7)	Reference		Reference	
	T2	19 (6.7)	0.741 (0.420, 1.307)		0.638 (0.325, 1.255)	
	T3	19 (8.7)	0.932 (0.528, 1.645)		0.480 (0.219, 1.052)	
Spot urine ACR < 300 mg/g	T1	27 (10.8)	Reference	0.066	Reference	0.146
	T2	15 (5.6)	0.470 (0.250, 0.884)		0.577 (0.281, 1.186)	
	T3	11 (4.0)	0.333 (0.165, 0.671)		0.293 (0.118, 0.723)	
Spot urine ACR ≥ 300 mg/g	T1	22 (7.2)	Reference		Reference	
	T2	25 (8.7)	1.187 (0.669, 2.106)		0.938 (0.457, 1.924)	
	T3	17 (6.2)	0.797 (0.423, 1.501)		0.447 (0.182, 1.098)	

Note: Adjusted HR of 24 h UPE as a continuous variable for 6-point MACE is depicted. The model was adjusted for age and sex, age-adjusted Charlson comorbidity index, the main cause of CKD, smoking status, medication (ACEi/ARBs, diuretics, number of anti-HTN drugs, statins), WHR, SBP, hemoglobin, albumin, total calcium, phosphorus, fasting glucose, hs-CRP, eGFR, spot urine ACR, LVMI, and LVEF. Abbreviations: 24 h UPE, 24 h urinary phosphorus excretion; ACR, albumin-to-creatinine ratio; BMI, body mass index; CI, confidence interval; eGFR, estimated glomerular filtration rate; HR, hazard ratio; T1, 1st tertile; T2, 2nd tertile, T3, 3rd tertile.

## Data Availability

Not applicable.

## Supplementary Materials

The following are available online at https://www.mdpi.com/article/10.3390/nu15102267/s1, Table S1: Incidence of 6-point MACE by CKD stages at the baseline; Table S2: Summary of echocardiographic findings of study participants by 24 h UPE; Table S3: HRs for 3-point and 4-point MACE by 24 h UPE level; Table S4: HRs for study outcomes by 24 h UPE level after excluding the subjects at CKD stage 1; Table S5: HRs for study outcomes by 24 h UPE level after excluding the subjects at CKD stage 5; Table S6: HRs for 6-point MACE by 24 h UPE level with the changes in the covariates.
