# Supplementary material for: Urinary Phosphorus Excretion and Cardiovascular Outcomes in Patients with Pre-Dialysis Chronic Kidney Disease: The KNOW-CKD Study
Source: Nutrients. 2023 May 10;15(10):2267. doi: 10.3390/nu15102267 (PMC10220543; doi:10.3390/nu15102267)
Supplement: Supplementary file 1 [file nutrients-15-02267-s001.zip › nutrients-2323092-supplementary.pdf]

– Supplementary Materials –

Urinary Phosphorus Excretion and Cardiovascular Outcomes in Patients with Pre-Dialysis Chronic Kidney Disease: the KNOW-CKD Study

Sang Heon Suh [1,2], Tae Ryom Oh [1,2], Hong Sang Choi [1,2], Chang Seong Kim [1,2], Eun Hui Bae [1,2], Seong Kwon Ma [1,2], Kook-Hwan Oh [3], Young Youl Hyun [4], Suah Sung [5] and Soo Wan Kim [1,2,*] on behalf of the Korean Cohort Study for Outcomes in Patients With Chronic Kidney Disease (KNOW-CKD) Investigators

[1] Department of Internal Medicine, Chonnam National University Medical School, Gwangju 61469, Republic of Korea
[2] Department of Internal Medicine, Chonnam National University Hospital, Gwangju 61469, Republic of Korea
[3] Department of Internal Medicine, Seoul National University Hospital, Seoul 03080, Republic of Korea
[4] Department of Internal Medicine, Kangbuk Samsung Hospital, Sungkyunkwan University School of Medicine, Seoul 03181, Republic of Korea
[5] Department of Internal Medicine, Eulji Medical Center, Eulji University, Seoul 01830, Republic of Korea
* Correspondence: skimw@chonnam.ac.kr; Tel.: +82-62-220-6271

Table of Contents

Table S1. Incidence of 6-point MACE by CKD stages at the baseline

Table S2. Summary of echocardiographic findings of study participants by 24h UPE

Table S3. HRs for 3-point and 4-point MACE by 24h UPE level

Table S4. HRs for study outcomes by 24h UPE level after excluding the subjects at CKD stage 1

Table S5. HRs for study outcomes by 24h UPE level after excluding the subjects at CKD stage 5

Table S6. HRs for 6-point MACE by 24h UPE level with the changes in the covariates

Table S1. Incidence of 6-point MACE by CKD stages at the baseline

	CKD stages	Events, n (%)	*P* value
6-point MACE	Stage 1	6 (2.1)	0.003
	Stage 2	26 (8.2)	
	Stage 3a	23 (8.2)	
	Stage 3b	32 (8.7)	
	Stage 4	32 (9.1)	
	Stage 5	3 (2.9)	

Abbreviations: CKD, chronic kidney disease; MACE, major adverse cardiac event.

Table S2. Summary of echocardiographic findings of study participants by 24h UPE

	24h UPE			
	T1	T2	T3	*P* value
LVMI (g/m^2)	93.484 ± 24.663	93.507 ± 24.426	96.821 ± 25.886	0.048
E/e’	10.579 ± 4.417	9.975 ± 4.239	9.512 ± 3.191	<0.001
LVEF (%)	64.287 ± 6.145	64.155 ± 6.600	64.024 ± 5.712	0.758
LAD (mm)	37.533 ± 6.183	38.162 ± 5.731	39.031 ± 5.857	<0.001
RWMA	21 (3.7)	21 (3.8)	17 (3.0)	0.485
Valve calcification	80 (14.2)	43 (7.7)	37 (6.5)	< 0.001
PWT (mm)	9.243 ± 1.671	9.291 ± 1.505	9.609 ± 1.555	<0.001
IVWT (mm)	9.288 ± 1.826	9.395 ± 1.621	9.737 ± 1.725	<0.001
LVEDD (mm)	47.677 ± 4.466	48.386 ± 4.787	49.506 ± 4.208	<0.001
LVESD (mm)	29.639 ± 4.119	30.150 ± 4.497	30.975 ± 4.107	<0.001

Note: Values for categorical variables are given as number (percentage); values for continuous variables, as mean ± standard deviation or median [interquartile range]. Abbreviations: 24h UPE, 24-hour urinary phosphorus excretion; E/e’, ratio of the early transmitral blood flow velocity to early diastolic velocity of the mitral annulus; IVWT, interventricular wall thickness; LAD, left atrium diameter; LVEDD, left ventricular end-diastolic diameter; LVEF, left ventricular ejection fraction; LVESD, left ventricular end-systolic diameter; LVMI, left ventricular mass index; PWT, posterior wall thickness; RMWA, regional wall motion abnormality; T1, 1st tertile; T2, 2nd tertile; T3, 3rd tertile.

Table S3. HRs for 3-point and 4-point MACE by 24h UPE level

	24h UPE level	Events, n (%)	Model 1		Model 2		Model 3		Model 4	
			HR (95%CIs)	*P* value	HR (95%CIs)	*P* value	HR (95%CIs)	*P* value	HR (95%CIs)	*P* value
3-point MACE	T1	32 (5.7)	Reference		Reference		Reference		Reference	
	T2	23 (4.1)	0.669 (0.372, 1.203)	0.180	0.698 (0.400, 1.218)	0.205	0.662 (0.363, 1.207)	0.178	0.614 (0.329, 1.147)	0.126
	T3	21 (3.7)	0.487 (0.255, 0.929)	0.029	0.638 (0.352, 1.157)	0.139	0.562 (0.292, 1.079)	0.083	0.436 (0.213, 0.893)	0.023
4-point MACE	T1	39 (6.9)	Reference		Reference		Reference		Reference	
	T2	32 (5.7)	0.803 (0.481, 1.341)	0.402	0.793 (0.486, 1.294)	0.353	0.863 (0.508, 1.465)	0.585	0.829 (0.480, 1.431)	0.500
	T3	26 (4.5)	0.495 (0.275, 0.892)	0.019	0.655 (0.383, 1.120)	0.122	0.608 (0.333, 1.109)	0.105	0.492 (0.257, 0.942)	0.033

Note: Model 1, unadjusted model. Model 2, model 1 + adjusted for age, sex, Charlson comorbidity index, the main cause of CKD, smoking history, medication (ACEi/ARBs, diuretics, number of anti-HTN drugs, statins), WHR, and SBP. Model 3, model 2 + adjusted for hemoglobin, albumin, total calcium, phosphorus, total cholesterol, LDL-C, HDL-C, TG, fasting glucose, 25(OH)D, hs-CRP, eGFR and spot urine ACR. Model 4, model 3 + LVMI and LVEF at the baseline. Abbreviations: 24h UPE, 24-hour urinary phosphorus excretion; MACE, major adverse cardiac event; T1, 1st tertile; T2, 2nd tertile; T3, 3rd tertile.

Table S4. HRs for study outcomes by 24h UPE level after excluding the subjects at CKD stage 1

	24h UPE level	Events, n (%)	Model 1		Model 2		Model 3		Model 4	
			HR (95%CIs)	*P* value	HR (95%CIs)	*P* value	HR (95%CIs)	*P* value	HR (95%CIs)	*P* value
6-point MACE	T1	48 (9.4)	Reference		Reference		Reference		Reference	
	T2	40 (8.5)	0.802 (0.501, 1.281)	0.355	0.827 (0.533, 1.284)	0.398	0.833 (0.518, 1.341)	0.452	0.729 (0.440, 1.209)	0.221
	T3	28 (6.4)	0.525 (0.304, 0.906)	0.021	0.601 (0.364, 0.995)	0.048	0.477 (0.271, 0.842)	0.011	0.415 (0.227, 0.076)	0.004

Note: Model 1, unadjusted model. Model 2, model 1 + adjusted for age, sex, Charlson comorbidity index, the main cause of CKD, smoking history, medication (ACEi/ARBs, diuretics, number of anti-HTN drugs, statins), WHR, and SBP. Model 3, model 2 + adjusted for hemoglobin, albumin, total calcium, phosphorus, total cholesterol, LDL-C, HDL-C, TG, fasting glucose, 25(OH)D, hs-CRP, eGFR and spot urine ACR. Model 4, model 3 + LVMI and LVEF at the baseline. Abbreviations: 24h UPE, 24-hour urinary phosphorus excretion; MACE, major adverse cardiac event; T1, 1st tertile; T2, 2nd tertile; T3, 3rd tertile.

Table S5. HRs for study outcomes by 24h UPE level after excluding the subjects at CKD stage 5

	24h UPE level	Events, n (%)	Model 1		Model 2		Model 3		Model 4	
			HR (95%CIs)	*P* value	HR (95%CIs)	*P* value	HR (95%CIs)	*P* value	HR (95%CIs)	*P* value
6-point MACE	T1	47 (9.4)	Reference		Reference		Reference		Reference	
	T2	42 (7.8)	0.753 (0.473, 1.197)	0.230	0.805 (0.522, 1.242)	0.327	0.853 (0.535, 1.359)	0.503	0.769 (0.469, 1.260)	0.297
	T3	30 (5.3)	0.405 (0.234, 0.701)	0.001	0.565 (0.346, 0.923)	0.023	0.482 (0.278, 0.836)	0.009	0.386 (0.211, 0.705)	0.002

Note: Model 1, unadjusted model. Model 2, model 1 + adjusted for age, sex, Charlson comorbidity index, the main cause of CKD, smoking history, medication (ACEi/ARBs, diuretics, number of anti-HTN drugs, statins), WHR, and SBP. Model 3, model 2 + adjusted for hemoglobin, albumin, total calcium, phosphorus, total cholesterol, LDL-C, HDL-C, TG, fasting glucose, 25(OH)D, hs-CRP, eGFR and spot urine ACR. Model 4, model 3 + LVMI and LVEF at the baseline. Abbreviations: 24h UPE, 24-hour urinary phosphorus excretion; MACE, major adverse cardiac event; T1, 1st tertile; T2, 2nd tertile; T3, 3rd tertile.

Table S6. HRs for 6-point MACE by 24h UPE level with the changes in the covariates

	24h UPE level	Model 5		Model 6		Model 7		Model 8	
		HR (95%CIs)	*P* value	HR (95%CIs)	*P* value	HR (95%CIs)	*P* value	HR (95%CIs)	*P* value
6-point MACE	T1	Reference		Reference		Reference		Reference	
	T2	0.795 (0.488, 1.296)	0.358	0.735 (0.453, 1.191)	0.735	0.616 (0.323, 1.176)	0.142	0.719 (0.445, 1.163)	0.179
	T3	0.427 (0.232, 0.788)	0.006	0.369 (0.203, 0.669)	0.001	0.394 (0.186, 0.836)	0.015	0.367 (0.203, 0.662)	0.001

Note: Model 5, model 4 + BMI. Model 6, DBP, instead of SBP in model 4. Model 7, model 4 + iPTH. Model 8, eliminating total cholesterol from model 4.

Abbreviations: 24h UPE, 24-hour urinary phosphorus excretion; CI, confidence interval; HR, hazard ratio; MACE, major adverse cardiac event; T1, 1st tertile; T2, 2nd tertile; T3, 3rd tertile.
